# Diversity and Host Blood Meal Analysis of *Culicoides* (Diptera: Ceratopogonidae) from Laos

**DOI:** 10.3390/insects17060647

**Published:** 2026-06-18

**Authors:** Ronnalit Mintara, Wannachai Wannasingha, Chavanut Jaroenchaiwattanachote, Waraporn Jumpato, San Namtaku, Khamla Inkhavilay, Isara Thanee, Pairot Pramual

**Affiliations:** 1 Department of Biology, Faculty of Science, Mahasarakham University, Kantharawichai 44150, Thailand; ronnalitmintara@gmail.com (R.M.); waraporn.a2536@gmail.com (W.J.); isara.th@msu.ac.th (I.T.); 2 Center of Excellence in Biodiversity Research, Mahasarakham University, Kantharawichai 44150, Thailand; wannachai.w@msu.ac.th; 3 Biomedical Science Research Unit, Mahasarakham University, Mueang Mahasarakham 44000, Thailand; chavanut.j@msu.ac.th; 4 Department of Science and Mathematics, Faculty of Science and Health Technology, Kalasin University, Kalasin 46230, Thailand; san.na@ksu.ac.th; 5 Center of Excellence in Biodiversity, National University of Laos, Vientiane 7322, Laos; khamla.inkhavilay@nuol.edu.la

**Keywords:** biting midge, DNA barcode, insect vector

## Abstract

Many biting midge species of the genus *Culicoides* Latreille are significant pests and vectors that transmit diverse parasites to humans and other animals. However, knowledge of these hematophagous insects in Laos remains limited, with the most recent information reported more than four decades ago. In this study, we investigated *Culicoides* species diversity, DNA barcoding and host blood sources using specimens collected across seven provinces in northern, central, and southern Laos. A total of 4592 specimens were collected, comprising 3095 females and 1497 males. Morphological identification, complemented by DNA barcode analysis, revealed 26 species (24 named and 2 unnamed), including five (three named and two unnamed) new country records. *Culicoides peregrinus* was the most abundant species, representing 25.7% (1179 individuals), followed by *C. oxystoma* at 23.8% (1093 individuals), and *C. arakawae*/*C. mahasarakhamense*, which together comprised 18.5% (849 individuals) of the total specimens. DNA barcode analysis demonstrated that this genetic marker is effective for species identification of *Culicoides* in Laos. Of the 115 COI sequences, 103 (89.6%) were successfully matched with conspecifics in the BOLD database. Cryptic genetic diversity was detected in three species, *C. clavipalpis*, *C. palpifer*, and *C. huffi*, with two, two, and three divergent lineages, respectively. Host blood meal analysis revealed that chickens and domestic water buffalo were the most common blood sources for the investigated *Culicoides* species. These findings provide important baseline information for future studies on the pest and vectorial roles of *Culicoides* in Laos.

## Simple Summary

Understanding biodiversity, along with rapid and accurate species identification, is essential for monitoring and surveillance of pest and vector species. Biting midges of the genus *Culicoides* are significant pests and vectors that transmit a wide range of pathogens, including viruses, bacteria, protozoa, and filarial nematodes, which cause diseases in humans and other animals. In this study, we explored *Culicoides* species diversity, applied DNA barcoding, and identified host blood sources from specimens collected across seven provinces of Laos. In total, 26 taxa (24 named and two unnamed species) were identified based on morphological characteristics complemented by DNA barcode data. *Culicoides peregrinus*, *C. oxystoma*, and *C. arakawae*/*C. mahasarakhamense* were among the most common species, together accounting for 68% of the collected specimens. Molecular identification of host blood sources revealed that these biting midges feed on chicken, domestic water buffalo, cattle, Muscovy duck, and another bird species, the scaly-breasted munia (*Lonchura punctulata*).

## 1. Introduction

Biting midges of the genus *Culicoides* Latreille (Diptera: Ceratopogonidae) comprise 1347 species recorded worldwide [1]. These hematophagous insects are significant pests and vectors that transmit a wide range of parasites to humans and other animals. *Culicoides* species are known to transmit viruses, protozoa, and filarial nematodes that cause important diseases, including oropouche fever and mansonellosis in humans, bluetongue disease in ruminants, African horse sickness in equids, and leucocytozoonosis in poultry and wild birds [2]. Outbreaks of diseases such as bluetongue and African horse sickness can result in substantial economic losses due to their impact on economically important livestock [3,4].

Knowledge of vector biodiversity is essential for a comprehensive understanding of disease epidemiology [5]. However, research on *Culicoides* in Laos remains limited compared to neighboring countries such as Thailand [6,7,8,9] and the China–Laos border region (Yunnan Province) [10,11,12]. The most recent comprehensive study in Laos dates back to 1985, when Howarth [13] provided a species list, identification keys, morphological descriptions, and biological information for 66 species recorded in the country. Since then, there have been no further reports on *Culicoides* in Laos. This uneven geographic knowledge of medically important insect vectors may pose risks to both public health and the economy in Laos and neighboring countries [14].

In this study, we surveyed *Culicoides* diversity across seven provinces in Laos, covering northern, central, and southern regions. Species diversity was assessed using both morphological characteristics and molecular approaches based on mitochondrial cytochrome c oxidase subunit 1 (COI) barcoding sequences. Traditional morphological identification of *Culicoides* is challenging due to their small size (1.0–2.5 mm) [2] and the limited diagnostic characters available to distinguish closely related species [15,16,17]. Numerous studies have demonstrated that DNA barcoding is a valuable complementary tool for accurate species identification, thereby enhancing our understanding of *Culicoides* biodiversity [18,19,20,21]. Additionally, we employed molecular techniques to identify host blood sources of *Culicoides* in Laos. Knowledge of vertebrate host preferences in hematophagous insects is crucial for assessing their vectorial capacity and understanding disease transmission dynamics [22,23]. The findings of this study provide essential baseline data to support future research on the biodiversity and vector roles of *Culicoides* in Laos.

## 2. Materials and Methods

### 2.1. Specimen Collection and Identification

**Figure 1:**
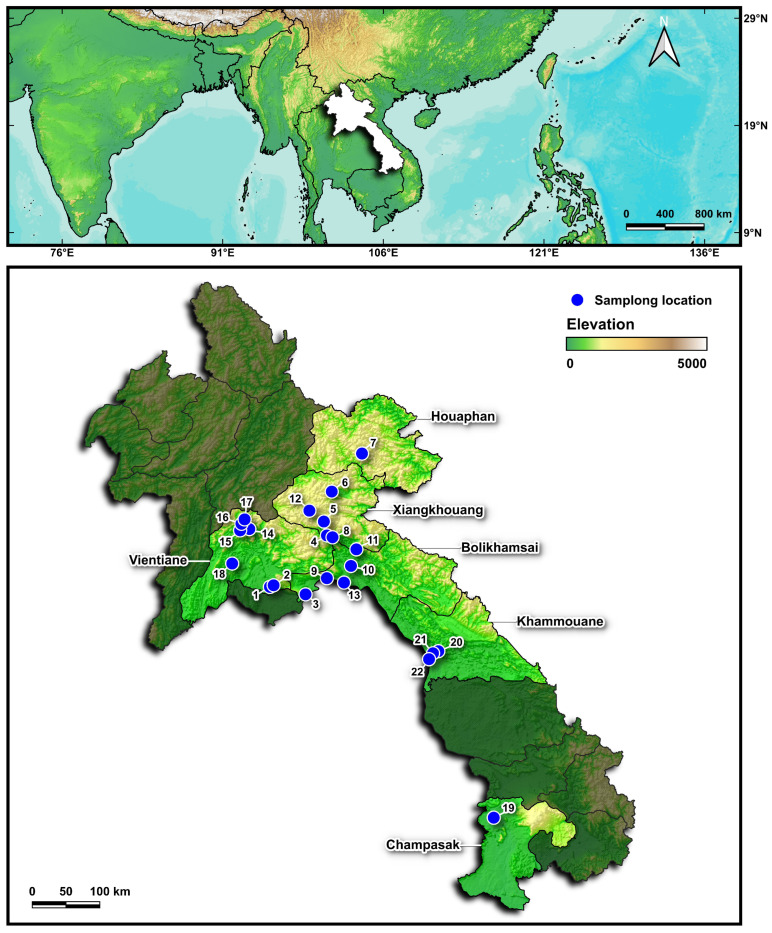
Map showing the geographical location of Laos (white area in the **top** panel) (retrieved from the Humanitarian Data Exchange (HDX) (https://data.humdata.org/dataset/cod-ab-lao) (accessed on 18 March 2026) and the study areas in seven provinces and sampling locations of *Culicoides* in Laos (**bottom** panel) (obtained from the EarthEnv project (https://www.earthenv.org/topography) (accessed on 18 March 2026). Detailed information for each sampling site is provided in Table 1.

Biting midge specimens were collected during 30 sampling events at 22 locations across seven provinces of Laos between August 2024 and October 2025 (Table 1; Figure 1). All sampling locations were near animal pens within villages. The methods employed were as follows: (i) CDC light traps. Specimens were collected using six CDC-style traps: one BG-Pro (Biogents, Regensburg, Germany) trap and five ABC Basic Light Traps (Clarke, St. Charles, IL, USA). Each trap was equipped with a black light (12 V, 65 W) and a non-filter catch bag as the collection container. The traps were placed about 50 m apart and operated overnight. (ii) Sweep netting. Adult midges were collected by three people sweeping nets through the air around the sampling sites. The sweep net had a hoop diameter of 39 cm and a three-part telescopic handle with a total extended length of 120 cm. Sampling was conducted for approximately one hour at each site. Collections began early in the morning (06:00 a.m.), with about one hour spent collecting.

Among the 22 sampling sites, 15 samples were taken using only sweep nets, and 5 were taken using only light traps. At one location, “Ban Na keo (2)” (Table 1), specimens were collected using both methods.

Collected specimens were preserved in 80% ethanol and stored at −20 °C until further analysis. Species identification was conducted based on morphological characteristics, using available taxonomic keys and descriptions for biting midges from Laos, Southeast Asia, and the *Culicoides* wing pictorial atlas [13,24,25].

### 2.2. Molecular Study

Representative specimens of each morphologically identified species were selected for DNA barcoding analysis. The head, wings and legs of specimen used for molecular analysis were removed and preserved in 80% ethanol and stored at −20 °C. The remaining body parts were used for DNA extraction using the GF-1 Nucleic Acid Extraction Kit (Vivantis Technologies Sdn. Bhd., Subang Jaya, Malaysia), following the manufacturer’s protocol. Extracted DNA was stored at −20 °C until further use. A fragment of approximately 650 bp of the mitochondrial cytochrome c oxidase subunit I (COI) gene was amplified using the primers LCO1490 and HCO2198 [26]. PCR conditions followed those described by Tangkawanit et al. [27]. PCR products were stained with Novel Juice (GeneDireX, Taoyuan, Taiwan, China) and visualized on 1% agarose gels. Successful amplicons were purified using the GF-1 AmbiClean (Gel & PCR) Kit (Vivantis Technologies Sdn. Bhd., Subang Jaya, Malaysia) and subsequently sequenced by ATCG Company Limited (Thailand Science Park, Pathum Thani, Thailand) using the same primers as for PCR amplification.

For host blood meal analysis, the mitochondrial cytochrome b (cyt b) gene was used as a genetic marker. Female specimens were examined under a stereomicroscope, and blood-fed individuals were selected for molecular identification of their host blood sources. DNA extraction was performed using the same protocol as described above. A fragment of approximately 300 bp of the cyt b gene was amplified using primers L14841 and H15149 [28]. PCR products were checked, purified, and sequenced following the same procedures as for COI analysis.

### 2.3. Data Analysis

COI sequences were checked and edited using the “Edit/View Sequence Files” function in MEGA X [29]. The COI sequences generated in this study (*n* = 115) were deposited in the NCBI GenBank database under accession numbers PZ319913-PZ320027. Species identification based on COI barcodes was performed using the “Identification Engine” in BOLD with the “Animal species-level library (Public + Private)” database and the “Rapid Species Search” option [30]. Identification was considered successful when sequences were assigned to the species level. Genetic relationships among COI sequences obtained in this study (*n* = 115) and those retrieved from the BOLD database (https://boldsystems.org/) for representative members of matched and nearest Barcode Index Numbers (BINs) (*n* = 135) were inferred using both neighbor-joining (NJ) and maximum likelihood (ML) methods. The NJ analysis was conducted in MEGA X with bootstrap support estimated from 1000 replications. The ML analysis was performed using the IQ-TREE web server [31,32]. The best-fit substitution model, selected based on the Bayesian Information Criterion, was GTR + F + I + G4. Branch support was assessed using ultrafast bootstrap with 1000 replications. Host blood meal identification was performed by comparing cyt b sequences from blood-fed specimens with sequences in the NCBI GenBank database using the Basic Local Alignment Search Tool (BLAST) (https://blast.ncbi.nlm.nih.gov/Blast.cgi) (accessed on 1 March 2026). Matches with ≥98% sequence similarity were considered to imply successful identification of host species.

## 3. Results

### 3.1. Species Diversity, Abundance and Occurrence

**Figure 2:**
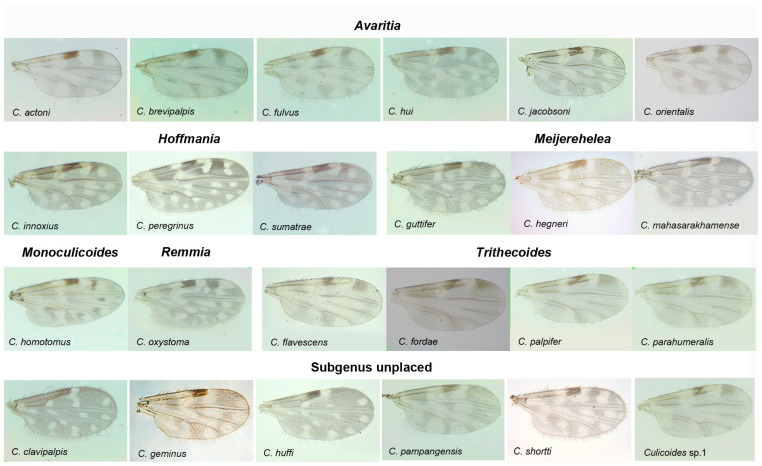
Photographs of female right wings of 24 species of *Culicoides* collected from seven provinces of Laos.

A total of 4592 specimens were collected from 22 locations across seven provinces of Laos between August 2024 and October 2025. Of these, 3095 were females (including 265 blood-fed individuals) and 1497 were males (Table 1). The majority of the collected specimens (4023 of 4592, or (87.6%)) were collected using the light trap method (Table 2). Morphological identification revealed 24 species, of which 18 were assigned to six subgenera, while six remained unplaced (Table 2; Figure 2). Among these species, 10 (*C. hui*, *C. jacobsoni*, *C. orientalis*, *C. sumatrae*, *C. hegneri*, *C. flavescens*, *C. fordae*, *C. palpifer*, *C. pamangensis* and *Culicoides* sp. 1) were only collected via the light trap method (Table 2).

DNA barcode identification using COI sequences was congruent with morphological identification for 103 of 115 (89.6%) specimens (Table 3). Incongruence between morphology and DNA barcoding was observed due to the absence of reference sequences (e.g., *C. jacobsoni*) and ambiguous matches for specimens of following species: two specimens morphologically determined as *C. innoxius* were matched with a BIN comprising two species *C. innoxius* and *C. sumatrae*; five specimens of *C. hegneri* were matched with a BIN comprising *C. hegneri*/*Culicoides* sp. All of *C. mahasarakhamense* were matched with conspecifics except one that was identified as *C. arakawae*.

Maximum intraspecific genetic divergence ranged from 0.16% in *C. brevipalpis* to 16.46% in *C. palpifer*. Most species exhibited low intraspecific divergence (<3%), although elevated divergence was also observed in *C. clavipalpis* (4.91%), *C. mahasarakhamense* (7.43%) and *C. huffi* (10.60%) (Table 3). Evidence of hidden diversity was detected, with 30 BINs identified among specimens representing 24 morphologically identified species. Five species were associated with multiple BINs, comprising *C. palpifer* (2 BINs), *C. clavipalpis* (2 BINs), *C. mahasarakhamense* (2 BINs), *C. huffi* (3 BINs), and *Culicoides* sp. 1 (2 BINs) (Table 3). One specimen morphologically identified as *Culicoides* sp. 1 was genetically highly different (>19%) from the others. Therefore, we treated this specimen as a separate species and refer to it here as *Culicoides* sp. 2. In *C. palpifer*, *C. clavipalpis*, and *C. huffi*, multiple BINs likely represent incomplete taxonomy (i.e., comprising multiple species that have not yet formally recognized). The two BINs associated with *C. mahasarakhamense* correspond to two closely related but distinct species (*C. mahasarakhamense* and *C. asakawae*). Integrating morphological and molecular data resulted in the recognition of a total of 26 species (Table 3).

Four species—*C. peregrinus*, *C. oxystoma*, *C. arakawae*/*C. mahasarakhamense*, and *C. huffi*—dominated the collections, accounting for 83.0% (3809 of 4591) of all specimens. The most abundant species was *C. peregrinus* (1179 specimens; 25.7%), followed by *C. oxystoma* (1093 specimens; 23.8%), *C. arakawae*/*C. mahasarakhamense* (849 specimens; 18.5%) and *C. huffi* (688 specimens; 15.0%) (Table 2). In terms of occurrence, *C. oxystoma*, *C. actoni*, and *C. arakawae*/*C. mahasarakhamense* were the most widespread species, being recorded in 17 (56.7%), 14 (46.7%), and 11 (36.7%) out of 30 collections, respectively. Most other species were found in fewer than 10 collections, and six species (*C. innoxius*, *C. hui*, *C. orientalis*, *Culicoides* sp. 1, *C. subflavescens*, and *Culicoides* sp. 2) were recorded only once (Table 1).

### 3.2. Phylogenetic Relationships

**Figure 3:**
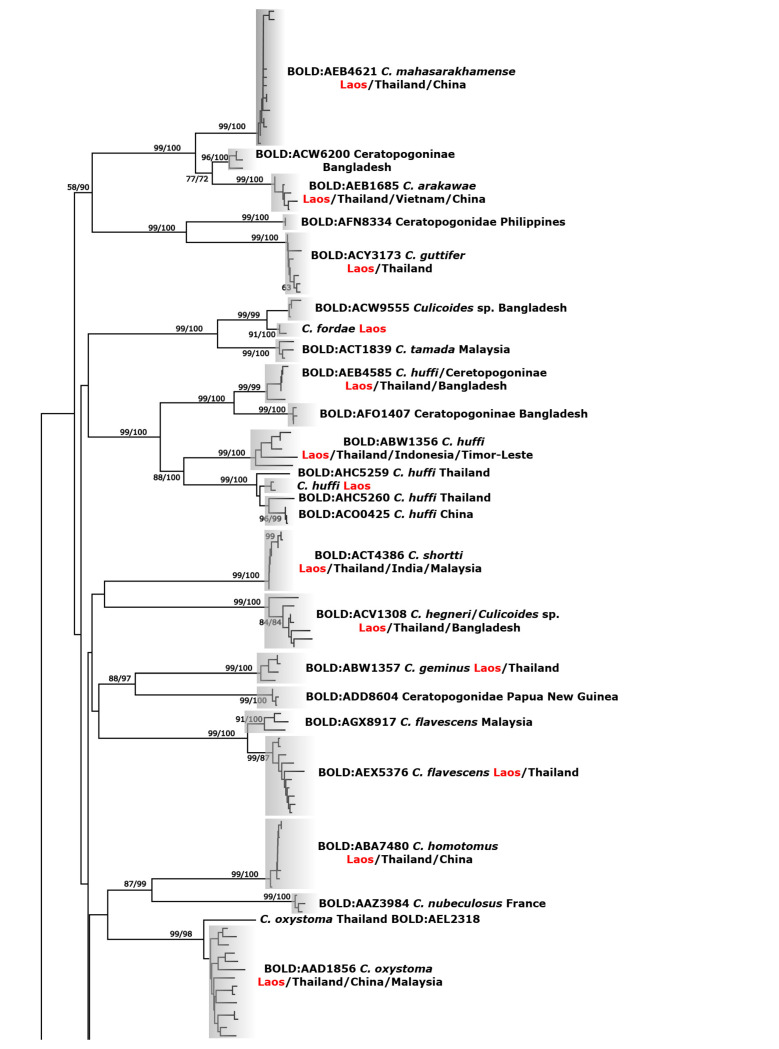

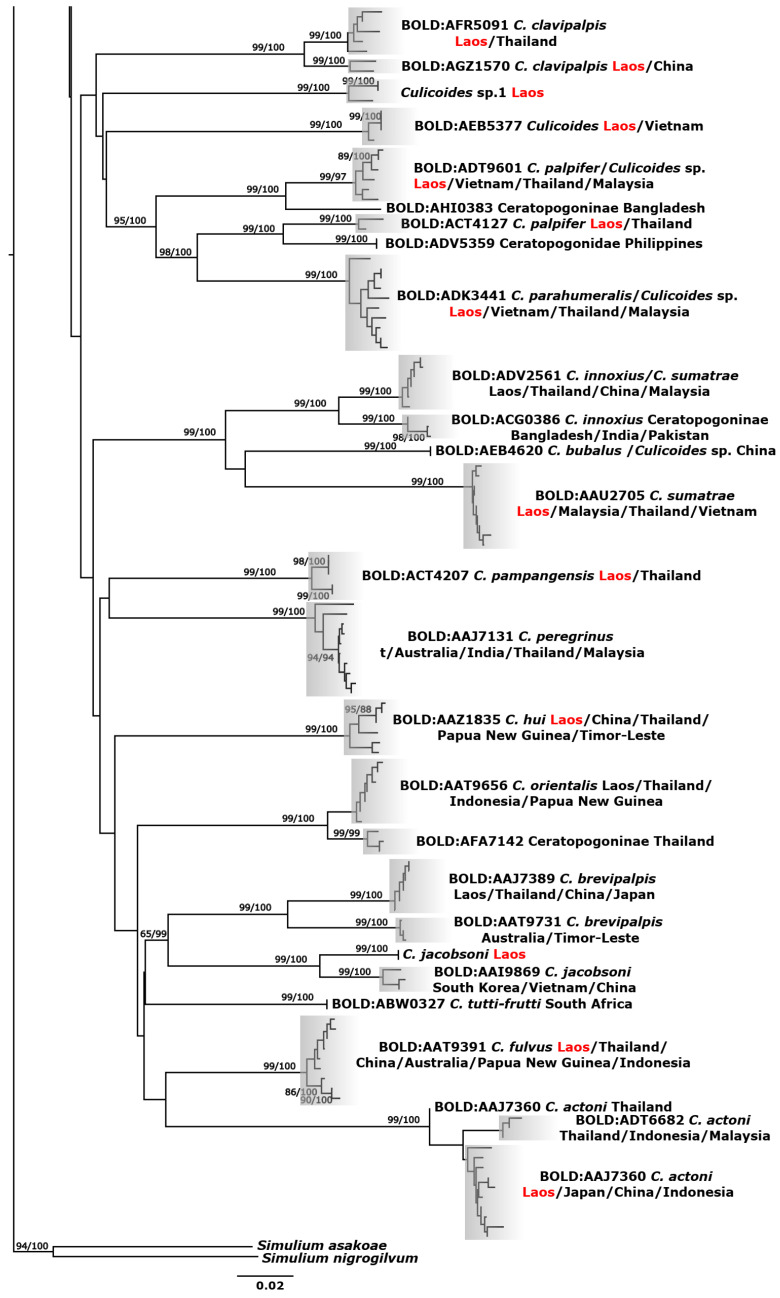
Neighbor-joining tree of 26 *Culicoides* species from Laos (red), inferred from mitochondrial cytochrome oxidase I (COI) sequences, together with conspecific and nearest-neighbor sequences retrieved from BOLD. For each species, country of origin, and BIN assignment are provided. Bootstrap values for NJ and ML analyses are shown above or near the branches. Details for each sequence are given in a Supplementary Table (Table S1).

Phylogenetic analyses based on neighbor joining (NJ) and maximum likelihood (ML) methods showed similar tree topologies. Therefore, only the NJ tree is presented here (Figure 3). The NJ tree showed that specimens from Laos clustered with conspecific sequences from other Asian countries and formed well-supported monophyletic clades (bootstrap > 90%) (Figure 3). However, three species—*C. clavipalpis*, *C. palpifer*, and *C*. *huffi*—exhibited deeply divergent lineages within their clades, consistent with their high intraspecific genetic divergence. Specimens of *C. clavipalpis* were separated into two distinct clades corresponding to different BINs, one clustering with specimens from Thailand and the other with specimens from China. Similarly, *C. palpifer* specimens were divided into two divergent lineages (BOLD:ADT9601 and BOLD:ACT4127). Specimens of *C. huffi* were separated into three distinct lineages that comprised five BINs (BOLD:AEB4585, BOLD:ABW1356, BOLD:AHC5259, BOLD:ACO0425 and BOLD:AHC5260).

### 3.3. Host Blood Meal Source Identification

A total of 97 blood-fed specimens representing 15 *Culicoides* species were selected for host identification. Of these, only 32 specimens were successfully sequenced (Table 4). Chickens (*Gallus gallus*) were the most common host, accounting for 66% (21 of 32) of identified blood meals. Species feeding on chickens included *C. clavipalpis*, *C. guttifer*, *C. mahasarakhamense*, and *C. huffi*. Additional avian hosts included scaly-breasted munia (*Lonchura punctulata*) and Muscovy duck (*Cairina moschata*), each detected in a single specimen of *C. huffi*. The second most common host was domestic water buffalo (*Bubalus bubalis*), representing 22% (7/32) of blood meals. Species feeding on this host included *C. actoni*, *C. hegneri*, *C. parahumeralis*, *C. oxystoma*, and *Culicoides* sp. 2. Additionally, single specimens of *C. innoxius* and *C. oxystoma* were found to have fed on cattle (*Bos indicus*).

## 4. Discussion

The results presented in this study represent the first report of *Culicoides* from Laos in more than four decades. The most recent study of this insect group was conducted by [13], who reported 62 named and 4 unnamed *Culicoides* species in Laos. Based on specimen collections from seven provinces, combined with morphological identification and DNA barcode analysis, we identified 24 named and 2 unnamed species. Among these, 21 species had been previously recorded in Laos [13,24], whereas five (three named and two unnamed) are newly recorded for the country. The newly recorded species include *C. homotomus*, *C. mahasarakhamense*, *C. pampangensis*, and two unnamed species (*Culicoides* sp. 1 and sp. 2). *Culicoides mahasarakhamense* was described in 2021 from Thailand [21], subsequent to the old report of *Culicoides* spp. from Laos by Howarth [13]. The two unnamed species (*Culicoides* sp. 1 and sp. 2) are documented for the first time in the present study. *Culicoides homotomus* and *C. pampangensis* are widely distributed in Southeast Asia [24]; therefore, their absence in the 1985 report by Howarth [13] is likely due to insufficient sampling.

The majority of specimens collected were females (3095 of 4592). This female-biased pattern is attributable to the substantially higher number of females than males captured using light traps (2846 vs. 1177). In contrast, sweep netting yielded a slightly higher number of males than females (320 vs. 249). A female bias in collections obtained using light traps has been reported previously [33,34]. This difference is likely related to sex-specific variation in responses to light [35].

Among the *Culicoides* species identified in Laos in the present study, at least four (*C. fulvus*, *C. guttifer*, *C. peregrinus*, and *C. oxystoma*) are known vectors of pathogens [2]. Other species may also serve as potential vectors; as disease-causing agents have been detected in them. For example, in neighboring Thailand, several parasites, including protozoa of the genera *Leucocytozoon*, *Plasmodium*, and *Trypanosoma*, have been detected in *C. huffi* and *C. mahasarakhamense* [7,36,37,38]. Also in Thailand, Bluetongue virus has been detected in *C. fulvus*, *C. orientalis*, and *C. oxystoma* [39]. In addition, *Leishmania martiniquensis* and *L. orientalis* have been detected in multiple *Culicoides* species, including *C. oxystoma*, *C. guttifer*, *C. orientalis*, *C. mahasarakhamense*, *C. innoxius*, *C. shortti*, *C. arakawae*, *C. sumatrae*, *C. jacobsoni*, *C. actoni*, *C. peregrinus*, and *C. fulvus* [40,41,42]. The presence of these *Culicoides* species in Laos therefore indicates a potential risk of diseases transmitted by these hematophagous insects, particularly for abundant vector species such as *C. peregrinus*, *C. mahasarakhamense* and *C. oxystoma*. Further investigations on the presence of pathogens in biting midge species in Laos are therefore critically important.

DNA barcode analysis demonstrated the high effectiveness of the COI gene sequences for species identification of *Culicoides*. Among the 115 sequences representing 24 morphologically identified species, 103 from 21 were successfully identified in BOLD. The remaining cases of unsuccessful identification included no match (*C. jacobsoni* and *Culicoides* sp. 1), ambiguous matches (*C. innoxius*/*C. sumatrae* and *C. hegneri*/*Culicoides* sp.), and cryptic species (*C. mahasarakhamense* and *C. arakawae*). Specimens of *C. jacobsoni* from Laos were genetically closest to conspecific sequences in the BOLD database but showed only 94% similarity; therefore, the identification engine did not assign them to the species level. Phylogenetic analyses further indicated that the Laotian specimens are distinct from *C. jacobsoni* recorded in other countries, suggesting that they may represent morphologically similar but genetically distinct species. Further studies are required to test this hypothesis.

Two specimens of *C. innoxius* from Laos were assigned to BIN BOLD:ADV2561, which includes specimens from Thailand, China, and Malaysia. This BIN contains both *C. innoxius* and *C. sumatrae*, although the majority (74 of 75 specimens) belong to *C. innoxius*. This suggests that the occurrence of *C. sumatrae* in this BIN may be due to misidentification, and indeed, the two species are morphologically very similar [24]. Specimens of *C. hegneri* from Laos were assigned to BIN BOLD:ACV1308, which includes only two specimens: one from Thailand and one unidentified specimen from Bangladesh. Notably, four BINs have been reported for *C. hegneri* based on only six specimens from two countries (Thailand and India), suggesting potential hidden diversity within this species that requires further investigation.

*Culicoides mahasarakhamense*, originally described from Thailand [21], is morphologically very similar to *C. arakawae*, with reliable differentiation requiring examination of the male paramere [21]. However, COI sequences clearly distinguish these two species, showing >3.9% sequence divergence. Our results highlight the usefulness of DNA barcoding in distinguishing morphologically similar species such as *C. mahasarakhamense* and *C. arakawae*. Although only *C. mahasarakhamense* was identified morphologically, DNA barcode data indicated that *C. arakawae* also occurs in Laos.

Cryptic genetic diversity was detected in three *Culicoides* species in Laos: *C. clavipalpis*, *C. palpifer*, and *C. huffi*. Such cryptic diversity has been widely reported in DNA barcoding studies of *Culicoides*. The presence of genetically divergent lineages within these species has also been reported in Thailand [8,43,44] and Malaysia [45]. For example, six BINs are reported for *C. clavipalpis* in BOLD. Specimens from Laos (*n* = 3) were assigned to two BINs: two specimens to BOLD:AFR5091 (shared with Thailand) and one specimen to BOLD:AGZ1570 (previously recorded from China). These BINs differ by >3.96% sequence divergence, suggesting that they may represent putative cryptic species. Further investigation integrating morphological characteristics with additional genetic markers, such as ITS2 sequences or other nuclear genes, is desirable to test this hypothesis.

Specimens identified as *C. palpifer* from Laos exhibited extremely high genetic divergence (maximum 16.46% p-distance) and matched two out of 11 BINs reported for this species in BOLD. The taxonomy of *C. palpifer* is complex, with multiple studies reporting high genetic diversity within morphologically identified specimens [44,45,46,47]. Similarly, *C. huffi* specimens from Laos were assigned to three BINs among the 10 reported in BOLD, covering a wide geographic range from Timor-Leste to China and Bangladesh. Given this wide distribution and high genetic divergence (up to 10.60%), these lineages may represent distinct species. Further morphological and molecular analyses using additional genetic markers are necessary to test this hypothesis [18].

Identifying host blood sources in hematophagous insects, particularly vector species, is essential for understanding vectorial capacity and disease epidemiology [22,23]. In this study, we report for the first time the vertebrate host blood sources of *Culicoides* in Laos. Among 97 blood-fed specimens used for the molecular identification of host blood sources, cyt *b* amplification was successful in only 42. This high rate of unsuccessful amplification may be related to several factors, including time elapsed since feeding. A more digested blood meal can reduce the success rate of PCR amplification because the vertebrate DNA in the blood meal degrades gradually over time [48]. Most of our blood-fed specimens (198 of 265) were collected using light traps, which were operated overnight before the specimens were preserved in ethanol. This extended period likely allowed for digestion of the blood meals, thereby reducing the quantity and quality of viable DNA and contributing to unsuccessful amplification. The DNA was successfully amplified but failed in sequencing, possibly due to the presence of blood from multiple vertebrate species [48].

Analysis of 32 successfully sequenced blood-fed specimens representing 10 species revealed five vertebrate host species. Chicken was the most common host (21 of 32, 66%), consistent with previous findings in Thailand [43]. However, a later study in Thailand reported domestic water buffalo as the most common host [8], likely reflecting differences in the *Culicoides* species examined or differences in local abundances of the two hosts.

Preliminary evidence from limited samples suggests that there is a degree of host preference among *Culicoides* species. Among the 10 species analyzed, six (*C. actoni*, *C. hegneri*, *C. parahumeralis*, *C. innoxius*, *C. oxystoma*, and *Culicoides* sp. 2) were mammalophilic, while four (*C. clavipalpis*, *C. huffi*, *C. mahasarakhamense*, and *C. guttifer*) were ornithophilic. These findings are consistent with previous studies [43]. Braverman et al. [49] proposed that mammalophilic species possess fewer (4–6) flagellomeres bearing sensilla coeloconica, whereas ornithophilic species possess more (12–13). Our results partially support this hypothesis: mammalophilic species exhibited 4–6 such flagellomeres, while two ornithophilic species (*C. mahasarakhamense* and *C. guttifer*) had 12. However, exceptions were observed, as *C. clavipalpis* and *C. huffi*—which feed on birds—had fewer sensilla [21,24]. Similar feeding patterns have been reported in Thailand [8,42]. However, because our results are based on a limited number of samples, further investigation from larger sample sizes and a more diverse range of species collected from diverse habitats is required.

## 5. Conclusions

In conclusion, this study provides the first report on *Culicoides* in Laos in over four decades. Among the 26 species identified, six are newly recorded for the country. DNA barcoding revealed cryptic genetic diversity in three species (*C. huffi*, *C. palpifer*, and *C. clavipalpis*), suggesting the presence of additional undescribed species. Further integrative taxonomic studies combining detailed morphological analyses with additional molecular markers are needed to clarify species boundaries. Host blood meal identification revealed that chickens and domestic water buffalo are the most common blood sources for the most abundant species (e.g., *C. oxystoma*, *C. huffi*, and *C. mahasarakhamense*). These *Culicoides* species are known or are considered to be potential vectors of pathogens affecting both domestic and wild animals. These findings highlight the need for further research on pathogen diversity and transmission dynamics in *Culicoides* populations in Laos.

## Figures and Tables

**Table 1:** Sampling location and number of male, female and blood-fed females of *Culicoides* collected from 22 locations in Laos between August 2024 and October 2025.

Location (Collection Methods; S, Sweep Net; T, CDC Light Trap)	Collection Date	Coordinates	Elevation (m)	Species	Number of Specimen (S/T)			
					Male	Female	Blood-Fed	Total
1. Ban Na Keo (1), Muang Thoulakhom, Vientiane (S)	4 August 2024	18.337433 N, 102.631475 E	168	*C. guttifer*	0	1	0	1
				*C. huffi*	1	1	0	2
				*C. mahasarakhamense*	0	1	4	5
				*C. oxystoma*	8	4	0	12
				*C. peregrinus*	24	36	46	106
				*C. shortti*	0	1	1	2
2. Ban Na Keo (2), Muang Thoulakhom, Vientiane (S, T)	20 September 2025	18.341101 N, 102.643600 E	176	*C. actoni*	0/6	0/16	1/11	1/33
	23 March 2025			*C. brevipalpis*	0/0	1/0	0/0	1
				*C. flavescens*	0/0	0/14	0/0	14
				*C. fordae*	0/1	0/18	0/0	19
				*C. fulvus*	0/0	1/13	0/0	14
				*C. geminus*	0/3	0/0	0/0	0/3
				*C. guttifer*	3/6	7/5	0	21
				*C. huffi*	0/6	15/61	0/5	87
				*C. laoensis*	0/6	0/40	0/2	48
				*C. mahasarakhamense*	147/40	47/46	3/5	288
				*C. oxystoma*	53/7	8/16	3/0	87
				*C. orientalis*	0/1	0/4	0/1	6
				*C. parahumeralis*	0/1	0/7	0/1	9
				*C. peregrinus*	30/166	12/55	2/1	266
				*C. shortti*	0/2	1/13	0/2	18
				*C. sumatrae*	0/4	0/10	0/0	14
				*C. pampangensis*	0/0	0/2	0/0	2
3. Muang Thaphabath, Borikhamxai (S)	4 January 2025	18.231388 N, 103.115357 E	172	*C. brevipalpis*	0	1	0	1
				*C. peregrinus*	1	0	0	1
				*C. oxystoma*	0	1	0	1
4. Muang Thathom, Xaisomboun (S)	5, 8 and 10 January 2025	19.037533 N, 103.407373 E	321	*C. actoni*	2	9	1	12
				*C. huffi*	0	0	1	1
				*C. mahasarakhamense*	0	0	1	1
				*C. oxystoma*	5	1	0	6
				*C. parahumeralis*	0	1	0	1
5. Muang Khoune, Xiangkhoang (S)	5, 8 January 2025	19.228120 N, 103.365560 E	995	*C. actoni*	3	5	0	8
	8 February 2025			*C. mahasarakhamense*	0	1	0	1
				*C. oxystoma*	1	1	0	2
6. Ban Yod Kuer Pu Muang, Muang Kham, Xiangkhoang (S)	6, 8 January 2025	19.637827 N, 103.473968 E	756	*C. actoni*	0	1	0	1
				*C. geminus*	0	0	1	1
				*C. mahasarakhamense*	0	1	0	1
				*C. oxystoma*	2	1	0	3
7. Ban Na Tor, Muang Houamuang, Houaphan (S)	7 January 2025	20.158222 N, 103.888923 E	1199	*C. actoni*	0	1	0	1
				*C. oxytoma*	0	1	0	1
				*C. shortti*	0	2	0	2
8. Ban Hau, Muang Thathom, Xaisomboun (S)	8 January 2025	19.022325 N, 103.452990 E	330	*C. oxystoma*	0	4	1	5
				*C. peregrinus*	3	1	0	4
9. Ban Nong Keun, Muang Thaphabath, Borikhamxai (S)	9 January 2025	18.452807 N, 103.406923 E	162	*C. brevipalpis*	1	0	0	1
	7 February 2025			*C. oxystoma*	0	1	0	1
				*C. peregrinus*	1	0	0	1
10. Muang Borikhanh, Borikhamxai (S)	8 February 2025	18.620325 N, 103.737733 E	174	*C. innoxius*	2	4	1	7
11. Ban Thasi, Muaung Thathom, Xaisomboun (S)	8 February 2025	18.847473 N, 103.811762 E	209	*C. oxystoma*	5	0	0	5
12. Ban Kang Yao, Muang Phonsavan, Xiangkhoang (S)	9 February 2025	19.376930 N, 103.168240 E	1119	*C. oxystoma*	0	1	0	1
13. Muang Pakxane, Borikhamxai (S)	11 February 2025	18.392865 N, 103.641953 E	159	*C. oxystoma*	0	1	0	1
14. Ban Pha Hom, Muang Vangvieng, Vientiane (S)	21 March 2025	19.125217 N, 102.344787 E	478	*C. actoni*	26	2	0	28
15. Ban Pha Chang (1), Muang Kasi, Vientiane (S)	21 March 2025	19.132047 N, 102.222658 E	430	*C. actoni*	1	0	0	1
				*C. clavipalpis*	0	1	0	1
				*C. shortti*	0	2	0	2
16. Muang Kasi, Vientiane (S)	22 March 2025	19.216227 N, 102.249352 E	412	*C. homotomus*	0	1	0	1
17. Ban Kaeo Chom Pu, Muang Kasi, Vientiane (S)	22 March 2025	19.257498 N, 102.280175 E	406	*C. actoni*	0	1	0	1
				*C. mahasarakhamense*	1	0	1	2
18. Ban Lao Kham (1), Muang Feuang, Vientiane (T)	22 March 2025	18.652842 N, 102.110338 E	227	*C. actoni*	1	3	0	4
				*C. fulvus*	0	1	0	1
				*C. guttifer*	0	2	0	2
				*C. hui*	0	3	0	3
				*C. huffi*	1	12	1	14
				*C. jacobsoni*	0	2	0	2
				*C. mahasarakhamense*	1	2	0	3
				*C. oxystoma*	1	5	0	6
				*C. parahumeralis*	2	8	0	10
				*C. shortti*	0	3	0	3
				*C. flavescens*	1	4	0	5
				*Culicoides* sp. 1	1	4	1	6
				*C. pampangensis*	0	1	0	1
19. Phonthong, Champasak (T)	7 March 2025	15.179200 N, 105.693850 E	107	*C. actoni*	0	1	0	1
				*C. guttifer*	0	1	0	1
				*C. huffi*	0	1	0	1
				*C. mahasarakhamense*	0	3	0	3
				*C. oxystoma*	1	0	0	1
				*C. parahumeralis*	1	0	0	1
				*C. shortti*	0	1	0	1
20. Xiangliab, Khammouan (T)	23 October 2025	17.457230 N, 104.935316 E	155	*C. actoni*	11	20	1	32
				*C. flavescens*	2	4	0	6
				*C. geminus*	1	5	3	9
				*C. guttifer*	4	2	3	9
				*C. huffi*	2	12	1	15
				*C. mahasarakhamense*	12	29	1	42
				*C. oxystoma*	23	30	0	53
				*C. parahumeralis*	3	8	0	11
				*C. peregrinus*	8	30	0	38
				*C. shortti*	4	24	1	29
21. Thakhek (1), Khammouan (T)	24 October 2025	17.428676 N, 104.862544 E	160	*C. actoni*	1	3	0	4
				*C. brevipalpis*	0	31	2	33
				*C. clavipalpis*	0	2	0	2
				*C. flavescens*	1	3	0	4
				*C. fulvus*	3	21	0	24
				*C. fordae*	3	0	0	3
				*C. geminus*	2	6	1	9
				*C. guttifer*	12	9	1	22
				*C. hegneri*	0	2	0	2
				*C. homotomus*	3	7	0	10
				*C. huffi*	72	188	36	296
				*C. jacobsoni*	0	1	0	1
				*C. mahasarakhamense*	215	71	13	299
				*C. oxystoma*	82	495	12	589
				*C. parahumeralis*	2	23	0	25
				*C. peregrinus*	91	318	10	419
				*C. shortti*	0	92	13	105
22. Thakhek (2), Khammouan (T)	25 October 2025	17.347937 N, 104.807317 E	166	*C. actoni*	5	10	7	22
				*C. clavipalpis*	0	0	3	3
				*C. flavescens*	0	6	0	6
				*C. fulvus*	2	18	0	20
				*C. geminus*	2	4	1	7
				*C. hegneri*	0	2	1	3
				*C. guttifer*	3	5	1	9
				*C. huffi*	98	152	22	272
				*C. mahasarakhamense*	120	60	24	204
				*C. oxystoma*	74	242	3	319
				*C. parahumeralis*	3	6	1	10
				*C. palpifer*	1	5	0	6
				*C. peregrinus*	56	285	3	344
				*C. shortti*	3	80	6	89
Total					1497	2830	265	4592

**Table 2:** Number of male, female and blood-fed females of *Culicoides* collected from Laos between August 2024 and October 2025.

Species	Male (S/T)	Female (S/T)	Blood-Fed (S/T)	Total (S/T)
*Avaritia* Fox				
*C. actoni* Smith	32/24	19/53	2/19	53/96
*C. brevipalpis* Delfinado	1/0	2/31	0/2	3/33
*C. fulvus* Sen & Das Gupta	0/5	1/53	0/0	1/58
*C. hui* Wirth & Hubert	0/0	0/3	0/0	0/3
*C. jacobsoni* Macfie	0/0	0/3	0/0	0/3
*C. orientalis* Macfie	0/1	0/4	0/1	0/6
*Hoffmania* Fox				
*C. innoxius* Sen & Das Gupta	2/0	4/0	1/0	7/0
*C. peregrinus* Kieffer	59/321	49/688	48/14	156/1023
*C. sumatrae* Macfie	0/4	0/10	0/0	0/14
*Meijerehelea* Wirth & Hubert				
*C. arakawae* (Arakawa)/*C. mahasarakhamense* Pramual et al.	148/388	50/211	9/43	207/642
*C. guttifer* (de Meijere)	3/25	8/24	0/5	11/54
*C. hegneri* Causey	0/0	0/4	0/1	0/5
*Monoculicoides* Khalaf				
*C. homotomus* Kieffer	0/3	1/7	0/0	1/10
*Remmia* Glukhova				
*C. oxystoma* Kieffer	74/188	24/788	4/15	102/991
*Trithecoides* Wirth and Hubert				
*C. flavescens* Macfie	0/4	0/31	0/0	0/35
*C. fordae* Lee	0/4	0/18	0/0	0/22
*C. palpifer* Das Gupta & Ghosh	0/1	0/5	0/0	0/6
*C. parahumeralis* Wirth & Hubert	0/13	1/57	0/2	1/72
subgenus unplaced				
*C. clavipalpis* Mukerji	0/0	1/2	0/3	1/5
*C. geminus* Macfie	0/8	0/15	1/5	1/28
*C. huffi* Causey	1/179	16/426	1/65	18/670
*C. pampangensis* Delfinado	0/0	0/3	0/0	0/3
*C. shortti* Smith & Swaminath	0/9	6/213	1/22	7/244
*Culicoides* sp. 1/*Culicoides* sp. 2	0/1	0/4	0/1	0/6
Total	320/1177	182/2648	67/198	569/4023

S, sweep net; T, CDC light trap.

**Table 3:** Number of specimens used for DNA barcode, intraspecific genetic divergence, identification results and nearest BIN in BOLD of biting midges from Laos.

Subgenus/ Species (*n*)	Intraspecific Genetic Divergence (Min.–Max.) (%)	BOLD Species Identification	BIN (*n*)	Sequence Identity with BOLD Sequences (%)	Nearest BIN (% Genetic Distance)	Nearest Taxon
*Avaritia*						
*C. actoni* (7)	0–1.58	*C. actoni*	BOLD:AAJ7360 (7)	98.06–100	BOLD:ADT6682 (1.46)	*C. actoni*
*C. brevipalpis* (2)	0–0.16	*C. brevipalpis*	BOLD:AAJ7389 (2)	98.16–100	BOLD:AAT9731 (6.81)	*C. brevipalpis*
*C. fulvus* (4)	0.16–0.63	*C. fulvus*	BOLD:AAT9391 (4)	98.72–100	BOLD:ABW0327 (10.63)	*C. tuttifrutti*
*C. hui* (2)	N/A	*C. hui*	BOLD:AAZ1835 (2)	97.60–100	BOLD:AEF9461 ^a^ (10.08)	N/A
*C. jacobsoni* (2)	0	N/A	N/A	94.50–94.82	BOLD:AAI9869 (3.18)	*C. jacobsoni*
*Hoffmania*						
*C. innoxius* (2)	0	*C. innoxius*/ *C. sumatrae*	BOLD:ADV2561 (2)	99.68–100	BOLD:ACG0386 (4.05)	*C. innoxius*
*C. peregrinus* (6)	0.16–0.79	*C. peregrinus*	BOLD:AAJ7131 (6)	99.05–100	BOLD:AEU8838 (11.22)	Ceratopogonidae
*C. sumatrae* (5)	0–0.63	*C. sumatrae*	BOLD:AAU2705 (5)	99.20–100	BOLD:AEB4620 (14.26)	*C. bubalus*
*Meijerehelea*						
*C. guttifer* (5)	0.32–1.11	*C. guttifer*	BOLD:ACY3173 (5)	99.01–100	BOLD:AFN8334 (8.17)	Ceratopogonidae
*C. hegneri* (5)	0.16–2.69	*C. hegneri*/*Culicoides* sp.	BOLD:ACV1308 (4)	98.51–99.67	BOLD:AFD7233 (1.83)	*Culicoides* sp.
			BOLD:AFD7233 (1)	97.19–99.50		
*C. mahasarakhamense* (15)	0–7.43	*C. mahasarakhamense*	BOLD:AEB4621 (14)	99.68–100	BOLD:ACW6200 (3.58)	*C. arakawae*
		*C. arakawae*	BOLD:AEB1685 (1)	99.84–100	BOLD:ACW6200 (2.87)	*C. arakawae*
*Monoculicoides*						
*C. homotomus* (6)	0–0.32	*C. homotomus*	BOLD:ABA7480 (6)	99.52–100	BOLD:AAZ3984 (9.82)	*C. nubeculosus*
*Oecacta*						
*C. orientalis* (3)	0–0.32	*C. orientalis*	BOLD:AAT9656 (3)	99.21–100	BOLD:AFA7142 (2.08)	Ceratopogoninae
*Remmia*						
*C. oxystoma* (10)	0.32–2.37	*C. oxystoma*	BOLD:AAD1856 (10)	97.94–100	BOLD:AEL2318 (1.66)	*C. oxystoma*
*Trithecoides*						
*C. flavescens* (7)	0.32–1.74	*C. flavescens*	BOLD:AEX5376 (7)	98.36–100	BOLD:AGX8917 (2.63)	*C. flavescens*
*C. fordae* (2)	0.32	*C. fordae*	BOLD:ACW9555 (2)	98.13–99.48	BOLD:ACT1839 (5.29)	*C. tamada*
*C. palpifer* (4)	0–16.46	*C. palpifer*	BOLD:ADT9601 (3)	98.41–100	BOLD:AHI0383 (5.77)	Ceratopogoninae
			BOLD:ACT4127 (1)	98.52–99.19	BOLD:ADV5359 (5.35)	*C. palpifer*
*C. parahumeralis* (6)	0–1.58	*C. parahumeralis*	BOLD:ADK3441 (6)	98.54–100	BOLD:AGX4349 ^a^ (10.90)	Ceratopogoninae
subgenus unplaced						
*C. clavipalpis* (3)	0–4.91	*C. clavipalpis*	BOLD:AFR5091 (2)	98.38–99.14	BOLD:AEA9303 ^a^ (3.74)	*C. clavipalpis*
			BOLD:AGZ1570 (1)	98.87	BOLD:AEA9303 ^a^ (1.46)	*C. clavipalpis*
*C. geminus* (1)	N/A	*C. geminus*	BOLD:ABW1357 (1)	99.84	BOLD:ADD8604 (10.59)	Ceratopogonidae
*C. huffi* (6)	0–10.60	*C. huffi*	BOLD:ABW1356 (2)	98.38–100	BOLD:AHC5259 (7.76)	*C. huffi*
			BOLD:ACO0425 (2)	98.18–99.01	BOLD:AHC5260 (1.54)	*C. huffi*
			BOLD:AEB4585 (2)	97.96–100	BOLD:AFO1407 (4.07)	*C. huffi*
*C. pampangensis* (3)	0	*C. pampangensis*	BOLD:ACT4207 (3)	98.68	BOLD:AEW2753 ^a^ (1.95)	*C. pampangensis*
*C. shortti* (5)	0	*C. shortti*	BOLD:ACT4386 (5)	99.68–100	BOLD:ADV8069 ^a^ (11.42)	Ceratopogoninae
*Culicoides* sp. 1 (4)	0–19.46	*C. albibasis*/ *C. flavescens*/ *C. rugulithecus*	BOLD:AEB5345 ^a^ (3)	98.02–99.68	N/A	N/A
		*Culicoides*	BOLD:AEB5377 (1)	99.35–99.84	BOLD:AHI0375 ^a^ (1.44)	*Culicoides* sp.

^a^ Private BIN, sequence data cannot access; N/A, data not available.

**Table 4:** Host blood source identification based on the cyt b sequences obtained from *Culicoides* blood meal.

*Culicoides* Species	Number of Blood-Fed Specimens	Number of Specimens Used for Host Blood Identification	Host Blood Identification (Number of Successful Specimens for Sequenced and Host Blood Source Identification)
*C. actoni*	21	11	Domestic water buffalo (*Bubalus bubalis*) (2)
*C. brevipalpis*	2	2	Unsuccessful PCR/Sequencing
*C. clavipalpis*	3	3	Chicken (*Gallus gallus*) (2)
*C. hegneri*	1	1	Domestic water buffalo *Bubalus bubalis* (1)
*C. guttifer*	5	5	Chicken (*Gallus gallus*) (3)
*C. geminus*	6	5	Unsuccessful PCR/Sequencing
*C. mahasarakhamense*	52	17	Chicken (*Gallus gallus*) (9)
*C. huffi*	66	14	Chicken (*Gallus gallus*) (6), Muscovy duck (*Cairina moschata*) (1), Scaly-breasted munia (*Lonchura punctulata*) (1)
*C. parahumeralis*	2	2	Domestic water buffalo (*Bubalus bubalis*) (2)
*C. innoxius*	1	1	Cattle (*Bos indicus*) (1)
*C. orientalis*	1	1	Unsuccessful PCR/Sequencing
*C. oxystoma*	19	12	Domestic water buffalo (*Bubalus bubalis*) (1), Cattle (*Bos indicus*) (1)
*C. peregrinus*	62	10	Unsuccessful PCR/Sequencing
*C. shortti*	23	12	Unsuccessful PCR/Sequencing
*Culicoides* sp. 2	1	1	Domestic water buffalo (*Bubalus bubalis*) (1)

## Data Availability

The sequences generated in this study were deposited in NCBI GenBank under accession numbers PZ319913–PZ320027. All other data and materials supporting this article are reported within the paper and its Supplementary Materials.

## Supplementary Materials

The following supporting information can be downloaded at https://www.mdpi.com/article/10.3390/insects17060647/s1, Table S1: Barcode Index Numbers (BINs), GenBank accession numbers or BOLD sequence IDs, and countries of origin for *Culicoides* COI sequences retrieved from BOLD and those collected in Laos (highlighted in red) used in the phylogenetic analyses.
