# Supplementary material for: Diversity and Host Blood Meal Analysis of Culicoides (Diptera: Ceratopogonidae) from Laos
Source: Insects. 2026 Jun 18;17(6):647. doi: 10.3390/insects17060647 (PMC13300096; doi:10.3390/insects17060647)
Supplement: Supplementary file 1 [file insects-17-00647-s001.zip › insects-4327104-supplementary.pdf]

Table S1. Barcode Index Numbers (BINs), GenBank accession numbers or BOLD sequence IDs, and countries of origin for *Culicoides* COI sequences retrieved from BOLD and those collected in Laos (highlighted in red) used in the phylogenetic analyses.

BIN ID	Species	Accession number/ BOLD sequence ID	Country
BOLD:AEB4621	*C. mahasarakhamense*	PQ340895	China
	C. mahasarakhamense	ON819812	Thailand
	C. mahasarakhamense	ON819839	Thailand
	C. mahasarakhamense	PZ319965	Laos
	C. mahasarakhamense	PZ319966	Laos
	C. mahasarakhamense	PZ319963	Laos
	C. mahasarakhamense	PZ319961	Laos
	C. mahasarakhamense	PZ319953	Laos
	C. mahasarakhamense	PZ319956	Laos
	C. mahasarakhamense	PZ319964	Laos
	C. mahasarakhamense	PZ319962	Laos
	C. mahasarakhamense	PZ319955	Laos
	C. mahasarakhamense	PZ319960	Laos
	C. mahasarakhamense	PZ319967	Laos
	C. mahasarakhamense	PZ319957	Laos
	C. mahasarakhamense	PZ319958	Laos
	C. mahasarakhamense	PZ319954	Laos
BOLD:ACW6200	Ceratopogoninae	GMBDB3605-23	Bangladesh
	Ceratopogoninae	GMBDC5989-24	Bangladesh
	Ceratopogoninae	GMBCF4239-15	Bangladesh
BOLD:AEB1685	*C. arakawae*	MW496166	Thailand
	C. arakawae	PQ340915	China
	C. arakawae	MZ189966	Thailand
	Ceratopogoninae	GMVNB21558-24	Vietnam
	C. arakawae	PZ319967	Laos
BOLD:AFN8334	Ceratopogonidae	GMPXB3653-23	Philippines
	Ceratopogonidae	GMPXB5012-23	
BOLD:ACY3173	*C. guttifer*	MW496179	Thailand
	C. guttifer	MW496178	Thailand
	C. guttifer	MZ191860	Thailand
	C. guttifer	PZ319944	Laos
	C. guttifer	PZ319943	Laos
	C. guttifer	PZ319945	Laos
	C. guttifer	PZ319946	Laos
	C. guttifer	PZ319947	Laos
BOLD:ACW9555	*Culicoides* sp.	GMBCG2385-15	Bangladesh
	Culicoides sp.	GMBDB5116-24	
	Culicoides sp.	GMBDB5198-24	
N/A	*C. fordae*	PZ319994	Laos
	C. fordae	PZ319995	Laos
BOLD:ACT1839	*C. tamada*	PQ775003	Malaysia
	C. tamada	ON002378	Thailand
	C. tamada	PQ775002	Malaysia
BOLD:AEB4585	Ceratopogoninae	GMBDG4616-24	Bangladesh
	C. huffi	PZ320015	Laos
	C. huffi	PZ320011	Laos
	C. huffi	MW496193	Thailand
	C. huffi	MW496226	Thailand
BOLD:AFO1407	Ceratopogoninae	GMBDC8151-24	Bangladesh

BIN ID	Species	Accession number/ BOLD sequence ID	Country
	Ceratopogoninae	GMBDA1389-23	Bangladesh
	Ceratopogoninae	GMBDC4705-24	Bangladesh
BOLD:ABW1356	*C. huffi*	KY441799	Thailand
	C. huffi	KY441778	Timor-Leste
	C. huffi	KY441780	Indonesia
	C. huffi	PZ320012	Laos
	C. huffi	PZ320014	Laos
BOLD:AHC5259	*C. huffi*	MZ191865	Thailand
BOLD:AHC5260	*C. huffi*	MZ191866	Thailand
N/A	*C. huffi*	PZ320010	Laos
	C. huffi	PZ320013	Laos
BOLD:ACO0425	*C. huffi*	PV810806	China
	C. huffi	KY441767	China
	C. huffi	KY441783	China
BOLD:ACT4386	*C. shortti*	ON002386	Thailand
	C. shortti	OK413625	India
	C. shortti	GMMNT181-18	Malaysia
	C. shortti	PZ320023	Laos
	C. shortti	PZ320022	Laos
	C. shortti	PZ320021	Laos
	C. shortti	PZ320020	Laos
	C. shortti	PZ320019	Laos
BOLD:ACV1308	*C. hegneri*	OR073944	Thailand
	Culicoides sp.	GMBCA3485-15	Bangladesh
	C. hegneri	PZ319948	Laos
	C. hegneri	PZ319949	Laos
	C. hegneri	PZ319950	Laos
	C. hegneri	PZ319951	Laos
	C. hegneri	PZ319952	Laos
BOLD:ABW1357	*C. geminus*	OR073899	Thailand
	C. geminus	OR073896	Thailand
	C. geminus	OR073892	Thailand
	C. geminus	PZ320009	Laos
BOLD:ADD8604	Ceratopogonidae	GMNGH1090-16	Papua New Guinea
	Ceratopogonidae	GMNGJ404-16	
	Ceratopogonidae	GMNGV929-16	
BOLD:AGX8917	*C. flavescens*	PQ776381	Malaysia
	C. flavescens	PQ776390	Malaysia
	C. flavescens	PQ776384	Malaysia
BOLD:AEX5376	*C. flavescens*	OR073920	Thailand
	C. flavescens	OR073921	Thailand
	C. flavescens	THAMA4850-22	Thailand
	C. flavescens	PZ319992	Laos
	C. flavescens	PZ319987	Laos
	C. flavescens	PZ319988	Laos
	C. flavescens	PZ319991	Laos
	C. flavescens	PZ319989	Laos
	C. flavescens	PZ319990	Laos
	C. flavescens	PZ319993	Laos
BOLD:ABA7480	*C. homotomus*	KY433504	China
	C. homotomus	PQ340913	China
	C. homotomus	MW496182	Thailand

BIN ID	Species	Accession number/ BOLD sequence ID	Country
	C. homotomus	PZ319968	Laos
	C. homotomus	PZ319969	Laos
	C. homotomus	PZ319970	Laos
	C. homotomus	PZ319971	Laos
	C. homotomus	PZ319972	Laos
	C. homotomus	PZ319973	Laos
BOLD:AAZ3984	*C. nubeculosus*	KJ624102	Slovakia
	C. nubeculosus	KP969014	France
	C. nubeculosus	MF594393	Turkiye
BOLD:AEL2318	*C. oxystoma*	MW496266	Thailand
BOLD:AAD1856	*C. oxystoma*	KT307835	India
	C. oxystoma	PQ776408	Malaysia
	C. oxystoma	MZ189958	Thailand
	C. oxystoma	KF528692	China
	C. oxystoma	PZ319978	Laos
	C. oxystoma	PZ319979	Laos
	C. oxystoma	PZ319977	Laos
	C. oxystoma	PZ319984	Laos
	C. oxystoma	PZ319983	Laos
	C. oxystoma	PZ319980	Laos
	C. oxystoma	PZ319981	Laos
	C. oxystoma	PZ319986	Laos
	C. oxystoma	PZ319982	Laos
	C. oxystoma	PZ319985	Laos
BOLD:AFR5091	*C. clavipalpis*	MZ189961	Thailand
	C. clavipalpis	OR073919	Thailand
	C. clavipalpis	OR073917	Thailand
	C. clavipalpis	PZ320007	Laos
BOLD:AGZ1570	*C. clavipalpis*	PV810799	China
	C. clavipalpis	PZ320006	Laos
N/A	*Culicoides* sp.1	PZ320024	Laos
	Culicoides sp.1	PZ320025	Laos
	Culicoides sp.1	PZ320026	Laos
BOLD:AEB5377	*Culicoides*	GMVNA13381-21	Vietnam
	Culicoides	GMVNA14141-21	Vietnam
	Culicoides	GMVNA13100-21	Vietnam
	Culicoides sp. 2	PZ320027	Laos
BOLD:ADT9601	*C. palpifer*	OR073963	Thailand
	Culicoides sp.	GMMSM822-18	Malaysia
	Culicoides sp.	GMVNA18228-21	Vietnam
	C. palpifer	PZ319996	Laos
	C. palpifer	PZ319997	Laos
	C. palpifer	PZ319998	Laos
BOLD:AHI0383	Ceratopogoninae	GMBDA3348-23	Bangladesh
BOLD:ACT4127	*C. palpifer*	ON002376	Thailand
	C. palpifer	PZ319999	Laos
BOLD:ADV5359	Ceratopogonidae	GMPMK2003-18	Philippines
		GMPXB352-23	Philippines
BOLD:ADK3441	*C. parahumeralis*	MH135786	China
	C. parahumeralis	PQ776368	Malaysia

**BIN ID**	**Species**	**Accession number/ BOLD sequence ID**	**Country**
	Culicoides sp.	GMVNA26745-23	Vietnam
	C. parahumeralis	THAMD22919-23	Thailand
	C. parahumeralis	PZ320001	Laos
	C. parahumeralis	PZ320002	Laos
	C. parahumeralis	PZ320004	Laos
	C. parahumeralis	PZ320000	Laos
	C. parahumeralis	PZ320003	Laos
	C. parahumeralis	PZ320005	Laos
BOLD:ADV2561	*C. innoxius*	GMMNK189-18	Malaysia
	C. innoxius	OM655214	China
	C. innoxius	OR073948	Thailand
	C. sumatrae	PV810812	China
	C. innoxius	PZ319931	Laos
	C. innoxius	PZ319930	Laos
BOLD:ACG0386	*C. innoxius*	GMBCE1223-15	Bangladesh
	C. innoxius	AGIRI210-17	India
	Ceratopogoninae	GMPKP3238-23	Pakistan
BOLD:AEB4620	*C. bubalus*	CUYUN533-19	China
	Culicoides sp.	MZ014371	China
BOLD:AAU2705	*C. sumatrae*	PQ776362	Malaysia
	C. sumatrae	MZ191875	Thailand
	C. sumatrae	CUYUN033-19	China
	C. sumatrae	GMVNA1884-21	Vietnam
	C. sumatrae	PZ319941	Laos
	C. sumatrae	PZ319939	Laos
	C. sumatrae	PZ319940	Laos
	C. sumatrae	PZ319938	Laos
	C. sumatrae	PZ319942	Laos
BOLD:ACT4207	*C. pampangensis*	ON002409	Thailand
	C. pampangensis	ON002407	Thailand
	C. pampangensis	PZ320016	Laos
	C. pampangensis	PZ320017	Laos
	C. pampangensis	PZ320018	Laos
BOLD:AAJ7131	*C. peregrinus*	CULIC560-11	Australia
	C. peregrinus	KT307848	India
	C. peregrinus	ON002397	Thailand
	C. peregrinus	PQ776380	Malaysia
	C. peregrinus	PZ319932	Laos
	C. peregrinus	PZ319933	Laos
	C. peregrinus	PZ319934	Laos
	C. peregrinus	PZ319935	Laos
	C. peregrinus	PZ319936	Laos
	C. peregrinus	PZ319937	Laos
BOLD:AAZ1835	*C. hui*	KT352494	Thailand
	C. hui	KT352232	China
	C. hui	KT352695	Papua New Guinea
	C. hui	KT352506	Timor-Leste
	C. hui	PZ319926	Laos
	C. hui	PZ319927	Laos
BOLD:AAT9656	*C. orientalis*	KT352329	Indonesia
	C. orientalis	KT352728	Papua New Guinea
	C. orientalis	ON002375	Thailand

BIN ID	Species	Accession number/ BOLD sequence ID	Country
	C. orientalis	PX226308	India
	C. orientalis	PZ319976	Laos
	C. orientalis	PZ319974	Laos
	C. orientalis	PZ319975	Laos
BOLD:AFA7142	Ceratopogoninae	THAMC7958-22	Thailand
	Ceratopogoninae	THAMC7669-22	Thailand
	Ceratopogoninae	THAMC8074-22	Thailand
BOLD:AAJ7389	*C. brevipalpis*	KT352195	China
	C. brevipalpis	ON002373	Thailand
	C. brevipalpis	KJ162965	Japan
	C. brevipalpis	OR073914	Thailand
	C. brevipalpis	PZ319921	Laos
	C. brevipalpis	PZ319920	Laos
BOLD:AAT9731	*C. brevipalpis*	KT352210	Timor-Leste
	C. brevipalpis	KT352617	Australia
	C. brevipalpis	KT352194	Australia
N/A	*C. jacobsoni*	PZ319928	Laos
	C. jacobsoni	PZ319929	Laos
BOLD:AAI9869	*C. jacobsoni*	KT352455	China
	C. jacobsoni	KF297817	South Korea
	C. jacobsoni	MK760174	Vietnam
BOLD:ABW0327	*C. tutti-frutti*	KJ162999	South Africa
	C. tutti-frutti	KJ162998	South Africa
BOLD:AAT9391	*C. fulvus*	KT352796	China
	C. fulvus	KT352315	Thailand
	C. fulvus	KT352271	Australia
	C. fulvus	KT352518	Papua New Guinea
	C. fulvus	KT352632	Indonesia
	C. fulvus	PZ319922	Laos
	C. fulvus	PZ319923	Laos
	C. fulvus	PZ319925	Laos
	C. fulvus	PZ319924	Laos
BOLD:AAJ7360	*C. actoni*	MW496131	Thailand
BOLD:ADT6682	*C. actoni*	GMIAA1311-18	Indonesia
	C. actoni	THAMJ11791-23	Thailand
	C. actoni	GMMSQ699-18	Malaysia
BOLD:AAJ7360	*C. actoni*	KT352426	Japan
	C. actoni	KT352567	China
	C. actoni	KT352700	Indonesia
	C. actoni	PZ319913	Laos
	C. actoni	PZ319919	Laos
	C. actoni	PZ319917	Laos
	C. actoni	PZ319916	Laos
	C. actoni	PZ319918	Laos
	C. actoni	PZ319914	Laos
	C. actoni	PZ319915	Laos

N/A, sequence similarity <98% to reference BIN members in BOLD.
